# Corneal Dellen

**DOI:** 10.5935/0004-2749.2023-0060

**Published:** 2024-05-09

**Authors:** Samara Barbara Marafon

**Affiliations:** 1 Hospital de Clínicas de Porto Alegre, Porto Alegre, RS, Brazil

**Figure 1.**
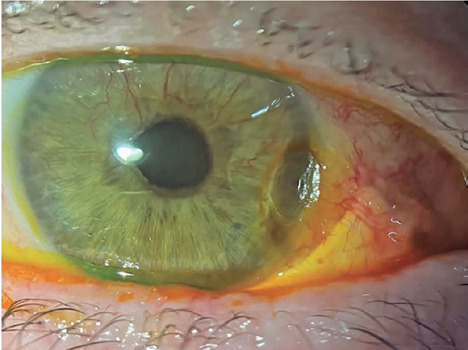
Corneal dellen following glaucoma device surgery

A 62-year-old woman complained of blurred vision 7 days postoperatively. She had undergone glaucoma surgery with Ahmed’s device due to neovascular secondary glaucoma in her only eye. Since day 1, she had been treated with gatifloxacin, prednisolone QID, and artificial tears. The glaucoma specialist prescribed eye lubricant and referred the patient to the cornea department. The image shows the right eye on postoperative day 10 (Figure 1). Corneal dellen is caused by tear film interruptions and local cornea dehydration^(1,2)^, usually adjacent to elevated areas, with limbal lesions and pterygium as the most common causes. Corneal dellen has been described following strabismus and filtering surgeries^(3,4)^. It may lead to corneal perforation if left untreated^(2)^.
